# Using sound to understand protein sequence data: new sonification algorithms for protein sequences and multiple sequence alignments

**DOI:** 10.1186/s12859-021-04362-7

**Published:** 2021-09-23

**Authors:** Edward J. Martin, Thomas R. Meagher, Daniel Barker

**Affiliations:** 1 School of Informatics, Informatics Forum, University of Edinburgh, 10 Crichton Street, Edinburgh, EH8 9AB UK; 2 Institute of Evolutionary Biology, School of Biological Sciences, University of Edinburgh, Charlotte Auerbach Road, The King’s Buildings, Edinburgh, EH9 3FL UK; 3 Centre for Biological Diversity, School of Biology, University of St Andrews, Sir Harold Mitchell Building, Greenside Place, St Andrews, KY16 9TH UK

**Keywords:** Sonification, Sequence analysis, Protein sequence, Multiple sequence alignment, Raspberry Pi, Sonic Pi, Algorithms, Qualitative research, Visualisation, Bioinformatics

## Abstract

**Background:**

The use of sound to represent sequence data—sonification—has great potential as an alternative and complement to visual representation, exploiting features of human psychoacoustic intuitions to convey nuance more effectively. We have created five parameter-mapping sonification algorithms that aim to improve knowledge discovery from protein sequences and small protein multiple sequence alignments. For two of these algorithms, we investigated their effectiveness at conveying information. To do this we focussed on subjective assessments of user experience. This entailed a focus group session and survey research by questionnaire of individuals engaged in bioinformatics research.

**Results:**

For single protein sequences, the success of our sonifications for conveying features was supported by both the survey and focus group findings. For protein multiple sequence alignments, there was limited evidence that the sonifications successfully conveyed information. Additional work is required to identify effective algorithms to render multiple sequence alignment sonification useful to researchers. Feedback from both our survey and focus groups suggests future directions for sonification of multiple alignments: animated visualisation indicating the column in the multiple alignment as the sonification progresses, user control of sequence navigation, and customisation of the sound parameters.

**Conclusions:**

Sonification approaches undertaken in this work have shown some success in conveying information from protein sequence data. Feedback points out future directions to build on the sonification approaches outlined in this paper. The effectiveness assessment process implemented in this work proved useful, giving detailed feedback and key approaches for improvement based on end-user input. The uptake of similar user experience focussed effectiveness assessments could also help with other areas of bioinformatics, for example in visualisation.

## Background

Using sound to represent data and exploit features of our psychoacoustic intuitions has a long history in scientific research. Galileo used humanity’s inherent sense of beat to measure the acceleration of balls down an inclined plane [1]. Hans Geiger and Walther Muller created a counter to monitor levels of local radiation without the need to check a dial [2]. Contemporary advertising research notes that radio adverts can remain effective when we pay them no attention [3].

Sonification is the modern term for the use of non-verbal sound to convey information [4]. In practice sonification augments the data visualisation approach to include sound, which can be used instead of or alongside visual information. Unlike many static visualisations, any display including sound will necessarily include a temporal aspect. Parameter-mapping sonification (PMSon) maps data features to sound synthesis parameters, for example monitoring a patient’s oxygen saturation via the pitch of a heart rate monitor’s beeping sound [5]. PMSons can utilise the multidimensional nature of sound to convey multivariate data, however the variety of mapping possibilities pose challenges of consistency and comprehensibility. Finding the balance between intuitive, pleasant, and precise sound is key for PMSon design [6].

As new technologies (e.g., single-cell or single-molecule DNA sequencing) produce genomic and proteomic data of rapidly increasing volume and complexity, the adoption of automated analysis methods (e.g., clustering, modelling, machine learning) within the life sciences is increasing. These automated analyses are not sufficient for knowledge discovery alone, and domain experts must inspect data to corroborate analyses. Data visualisation is the key method through which experts inspect their data. With increases in data, further innovations are needed in visualisation methods [7]. Multiple sequence alignments (MSAs) are one example of biological data where a new approach to representation may be promising. An MSA is a matrix created from a set of biological sequences, for example proteins. The sequences are modified by gap-insertion to create a multi-way, high scoring alignment. Biologists use MSAs to predict aspects of protein structure and identify protein domains; infer sequence homology and evolutionary relationships; discern protein disorder, function, and localisation; understand genomic rearrangements; and estimate evolutionary rates [8].

MSA visualisation software packages are essential tools for life scientists, however MSA visualisations often end up overloaded due to the complex properties of amino acids [9]. This complexity is often displayed using colour, which may be non-inclusive because ~ 5 percent of people are colour blind [10]. Generally, science relies heavily on visual resources which can be inaccessible to blind/partially sighted students unless presented in an alternative format [11]. Even for fully sighted users who are not colour blind, the amount of data can be too much to fit on a screen and may be confusing to navigate.

Previous research has demonstrated that molecular information can be sonified with positive results [12], with specific successes demonstrating DNA and protein sequence data [13, 14] and 3D protein structure [15]. We suggest that parameter-mapping sonification can be used to improve MSA visualisation, meet the need for innovation in protein sequence visualisation, and create high-quality low-outlay content for scientific communication.

Our aim is to harness the variety and power of human psychoacoustic intuition to the yoke of proteomic problems. This paper develops five sonification algorithms for representing protein sequence data using sound. These algorithms create sonifications which represent either single protein sequences or protein MSAs. We evaluate the effectiveness at conveying information of these algorithms by using end-users to assess the algorithms as aids to solving bioinformatics-inspired tasks through a questionnaire and a focus group.

## Methods

Two pieces of hardware were used for generating sonifications: a desktop computer running Scientific Linux 7.6 (Nitrogen) distribution with Linux kernel version 3.10.0.-957.12.1.el7.x86_64, and—because Sonic Pi is not supported on Scientific Linux—a Raspberry Pi running version 1.5 of the 4273pi variant of the Raspbian operating system [16].

**Fig. 1:**
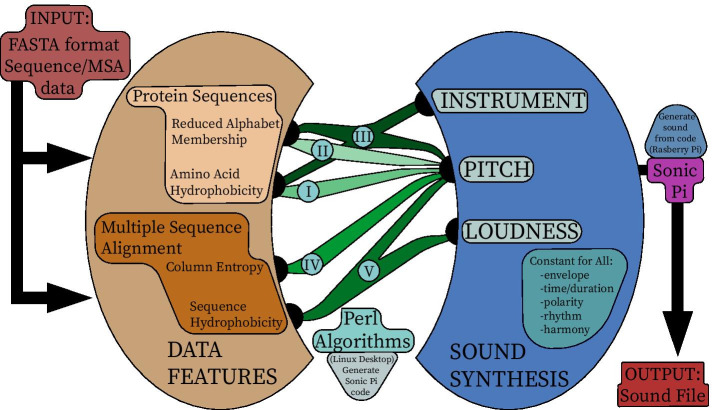
Illustration of the design process and data flow of the sonification algorithms. Each algorithm (I–V) is represented by a different path through the centre of the diagram, connecting the data feature to the sound synthesis parameter used to represent it. The data flows from the FASTA file input, through a Perl algorithm on a Linux machine, then through Sonic Pi on a Raspberry Pi machine, before coming out as a sound file. This illustration is inspired by [6] (their figure 15.1) which also includes a split between Data Features and Sound Synthesis

Perl (v5.16.3) and Sonic Pi (v3.1.0) were used. The flow of the data is illustrated in Fig. 1 and went as follows:

1. Input: Sequence(s) in Fasta format.
2. Using Perl on Linux desktop: Generate Sonic Pi code from sequence(s).
3. Using Sonic Pi on Raspberry Pi: Generate sound from code.
4. Output: sound file.

By building on and adapting a Perl script that was written to sonify DNA [17], we developed five parameter-mapping sonification algorithms (Algorithms I–V). The algorithm scripts can be found in our GitHub repository [18] and are archived in the supplementary materials online [19].

Each algorithm maps the amino acid position in the protein sequence or MSA to time, creating the metaphor of *playing* the sequences like sheet music or tablature, gramophones or LPs, cassette tapes or DATs, CDs or minidisks, music boxes or player pianos, depending on your age and background. These metaphors influenced our sound design.

We set the synthesizer’s envelope *attack* parameter to zero, giving immediate note onset and creating the sense of a regular beating pulse in the sonifications. We used different synthesizers to distinguish input data types, using *sine* synthesizers for protein sequence sonification (Algorithms I and II) and *saw* synthesizers for MSA sonification (Algorithms IV and V). Algorithm III used multiple synthesizers so did not follow this pattern. All synthesizers were chosen to convey pitch quickly and precisely, whilst maintaining the audio metaphor we sought, and being pleasant on the ears.

Our algorithms all use MIDI number to represent pitch, as is used in Sonic Pi. A MIDI number is an integer, typically between 0 and 127, with each number representing a half-note pitch in Western tonal music. The MIDI language for sending musical controls in real-time is the industry standard [20].

### Algorithm I: Protein sequence hydrophobicity

To sonify a single protein sequence, this sonification maps the 20 amino acids to 20 MIDI pitches based on their hydrophobicity, inspired by the work of Hayashi and Munakata [21]. Differing from their approach, we used much smaller pitch range to assist listeners in pattern recognition and derived our relative pitches from the Goldman, Engelman, and Steitz (GES) hydrophobicity scale, which has an experimental basis and uses a semitheoretical approach. GES is based on energetic considerations of residues in α-helices [22]. GES represents hydrophobicity with a score. The amino acids are thus ordered linearly.

**Fig. 2:**
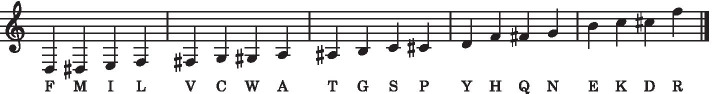
Musical score detailing our *Hydrophobicity Scale* developed from the data in Table 1. This mapping of amino acids to pitch is used in Algorithms I, III, and V

Working through the GES ordering from hydrophobic to hydrophilic, we started by mapping phenylalanine, the most hydrophobic residue, to the MIDI number 50. Each subsequent residue in the ordering was allocated a MIDI number equal to the previous amino acid’s MIDI number plus the increment in the GES score between the two amino acids. This was rounded up to the nearest integer to give a MIDI number, and also to ensure a one-to-one mapping. This mapping is detailed in Table 1 and Fig. 2.
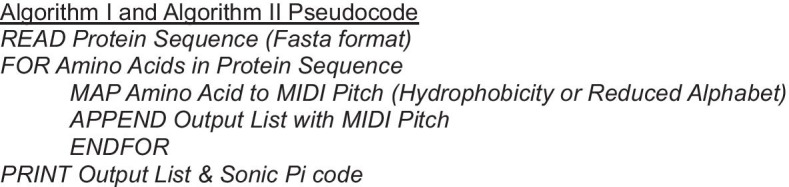

**Table 1:** Mapping from amino acids to MIDI pitch numbers using GES score [22]

Code	Amino-acid	GES score	Increment	MIDI number
*Amino acid pitch mapping*				
F	Phenylalanine	− 3.7		50
M	Methionine	− 3.4	0.3	51
I	Isoleucine	− 3.1	0.3	52
L	Leucine	− 2.8	0.3	53
V	Valine	− 2.6	0.2	54
C	Cysteine	− 2	0.6	55
W	Tryptophan	− 1.9	0.1	56
A	Alanine	− 1.6	0.3	57
T	Threonine	− 1.2	0.4	58
G	Glycine	− 1	0.2	59
S	Serine	− 0.6	0.4	60
P	Proline	0.2	0.8	61
Y	Tyrosine	0.7	0.5	62
H	Histidine	3	2.3	65
Q	Glutamine	4.1	1.1	66
N	Asparagine	4.8	0.7	67
E	Glutamate	8.2	3.4	71
K	Lysine	8.8	0.6	72
D	Aspartate	9.2	0.4	73
R	Arginine	12.3	3.1	77

Used in Algorithms I, III, and V

### Algorithm II: Protein sequence reduced alphabet

Here we take a different approach to the sonification of single protein sequences. It is inspired by the work of King and Angus [23], whose algorithm uses a reduced alphabet to simplify the 20 amino acids to a representation comprised of four letters. In our algorithm, each letter of the reduced alphabet represents a group of amino acids with similar hydrophobicity [24]. We allocated pitches to each of these groups and assigned them a MIDI pitch corresponding to the first 4 notes of a C major pentatonic scale. They were grouped as follows: FILVWY with MIDI pitch 67, ACGMP with MIDI pitch 64, KQST with MIDI pitch 62 and DEHNR with MIDI pitch 60.

### Algorithm III: Protein sequence hydrophobicity and reduced alphabet

To sonify a single protein sequence incorporating both the detail of Algorithm I and the broader resolution of Algorithm II, we combined the two approaches. We used the GES scale-based mapping of Algorithm I detailed in Table 1 and Fig. 2 to determine pitch. We incorporated Algorithm II by using a different *instrument* within Sonic Pi for each reduced alphabet group. The synthesizers were allocated as follows: FILVWY to *piano*; ACGMP to *sine*; KQST to *pluck*; and DEHNR to *tb303*.
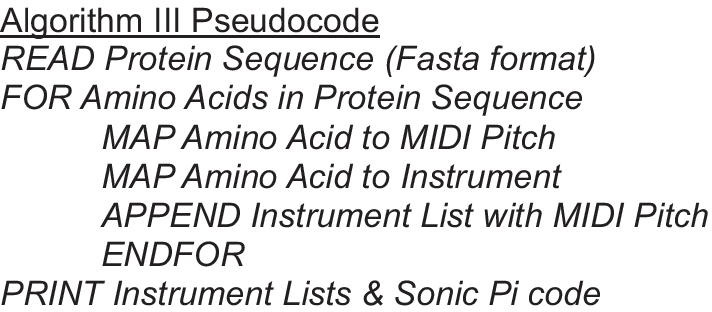


### Algorithm IV: MSA entropy

Our first approach to MSA sonification concerns higher level information, taking some inspiration from the PROMUSE software system [15]. Our algorithm gives a monophonic output, with each note representing a column of the MSA. The pitch of the note represents the level of conservation of the proteins at the corresponding position within the MSA, more variety in the column gives a higher pitch. Therefore, more high-pitched regions mean less conservation, and vice versa. We measured the variety of each column within the MSA by calculating its Shannon entropy, where a higher entropy corresponds to more columnar variety [25].

*H*_*i*_, the Shannon entropy for the *i*-th column, is defined as:

$$H_{i} = - \mathop \sum \limits_{J} p_{jk} \log_{2} \left( {p_{jk} } \right)$$

where $j_{k} \in J$ is the set of *k* unique amino acid symbols (including the gap character ‘-’) present in the *i*-th column, and

$$p_{jk} = \frac{{\tilde{j}_{k} }}{n}$$

where $\tilde{j}_{k}$ is the count of $j_{k}$ in the *i*-th column, and *n* is the number of proteins in the MSA.

This outputs a single value for each column of the MSA. To map these to MIDI numbers, we used a z-score standardisation of the set of all column entropy values. We then scaled this to a listenable range within the MIDI range 0–127, and took the floor value to give an integer. Our output data set $H^{*}$ of MIDI numbers is composed of transformed data points $H_{i}^{*}$ such that:

$$H_{i}^{*} = \frac{{H_{i} - \overline{H}}}{{s_{H} }} \cdot \,10 + 60$$

where *H*_*i*_ is the data point under transformation, and $\overline{H}$ and *s*_*H*_ are the mean and standard deviation (with division by n-1) of the set of all column entropies *H*. Adding 60 means that the pitches will be centred around middle C (MIDI number 60), and scaling by 10 creates a manageable spread of notes. These two figures are based on personal preference.
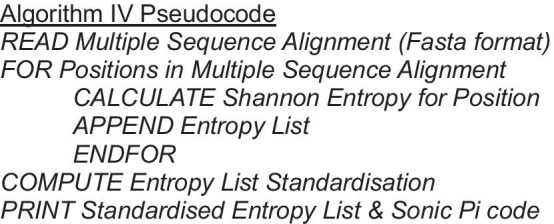


### Algorithm V: MSA hydrophobicity

This approach sonifies each row of the input MSA simultaneously, using the mapping described in Algorithm I, detailed in Table 1 and Fig. 2. This creates a polyphonic output using the *saw* synthesizer. If the same residue is present at the same position in multiple rows, then the volume of the note of the corresponding pitch increases. Gaps in the alignment do not sound. For example, a loud single note represents a consensus whereas a quiet single note represents a gap in most sequences. This contrasts to Algorithm I in several ways: the output is polyphonic, the *saw* synthesizer is used instead of the *sine* synthesizer, it takes MSA as input instead of a single protein sequence, and the volume changes to represent how many sequences have the same residue at that position in the MSA.
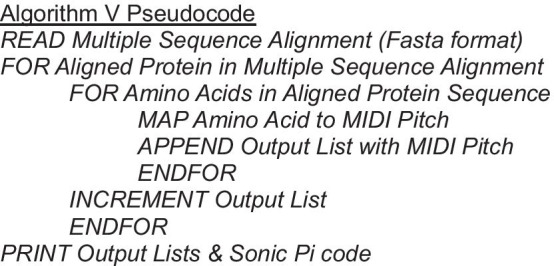


### Example sonifications

Examples of all five algorithms can be found at https://sonifyed.com/bmc-bioinformatics-2021.

Protein sonification (Algorithms I, II, and III) examples include transmembrane, globular, and disordered proteins, and a protein containing an amino acid repeat (AAR). MSA sonification (Algorithms IV and V) examples compare gappy and compact alignments of insulin and glyceraldehyde 3-phosphate dehydrogenase. The gappy and compact MSAs were made using the same input sequences and MUSCLE v3.8.31 [26]. A *gapopen* penalty of − 3 was used for compact MSAs and of + 1 for gappy MSAs.

### Assessment of effectiveness

The approach taken to assessing the effectiveness of the sonification algorithms focused on the user experience of bioinformatics researchers—the target end-users of the technology. It was predominantly qualitative and rooted in subjective phenomenological judgements. It comprised of an online questionnaire and a focus group session.

### Recruitment of participants

Participants were recruited using three mailing lists for Scottish bioinformatics researchers: *Ashworth Bioinformatics Club* (staff and postgraduate students, with an emphasis on those working in a single building at the University of Edinburgh), *Edinburgh Bioinformatics* (staff and postgraduate students across research institutions in Edinburgh and South East Scotland), and *NextGenBUG* (professionals across Scotland).

The sampling was non-random within these mailing lists. The expression of interest was the key factor contributing to inclusion in the sample—everyone who expressed interest was included. All respondents self-confirmed that they met our criteria of advanced biological knowledge.

### Questionnaire

The questionnaire centred on Algorithm I and Algorithm V. This allowed the assessment of one single protein sonification and one MSA sonification, while keeping cognitive workload for participants low to ensure quality responses. Participants completed tasks using the sonifications as tools. A PDF of the questionnaire is available via our website (https://sonifyed.com/bmc-bioinformatics-2021), via GitHub [18] and archived in our supplementary material [19]. Compared to the online form used in the survey, we have replaced the password-protected URL for sound files with a publicly available link, making this easy for readers to try themselves (although we are no longer collecting data).

#### Task 1

Amino acid repeats (AARs) are repeated sequences of amino acids found within proteins. They have particular roles in protein function and evolution, however they are poorly understood, and their identification is difficult, as it is not possible to define a uniform criterion for detecting and verifying various repeat patterns [27].

In the first task participants were asked to identify an amino acid repeat using a sonification produced using Algorithm I. It was eight letters long and repeated four times in tandem. The whole protein sequence had length 253. Participants were informed that the protein contained a “a short (< 20 letters) amino acid motif, or word, repeated four times”. They were also supplied with a basic visualisation of the protein sequence in the form of an image taken from MView [28], with no use of colour schemes.

#### Task 2

Protein domains are distinct units in a protein. They are often responsible for a particular function or interaction. The same domain can be found within many different proteins, and typically performs the same function in each. MSAs are one of the most widely used methods for identifying and assessing the conservation of protein domains [29].

In the second task participants were asked to identify three conserved domains within a sonification of an MSA of five protein sequences created using Algorithm V. All of the protein sequences used to create the MSA contained two examples of the SH3_1 conserved domain and one example of the SH2 conserved domain (PF00018 and PF00017). Participants were told that “this MSA contains three conserved domains (< 50 letters)” and were tasked with identifying them. They were also supplied with a visualisation in the form of an image from MView with no colour scheme.

#### Responses

After attempting each task, the participants were shown the sequence or multiple alignment with the region(s) of interest highlighted with brightly coloured bars (and in the case of the multiple sequence alignment, with the amino acids in the multiple alignment coloured) and were asked the following three questions

1. “Did the sonification file help you identify the {repeated motif}/{conserved domain}?”—with a “Yes/No” response.
2. “What was the best thing about the sonification?”—with a free text response.
3. “What was the worst thing about the sonification?”—with a free text response.

#### Workload

We evaluated the subjective mental workload of the more difficult second task using the NASA Task Load Index (TLX). It is a subjective, multi-dimensional, quantitative assessment tool that assesses the perceived workload of a task [30]. Independent researchers have demonstrated both the reliability and validity of the TLX [31, 32] and its frequent use in the literature [33].

The workload is divided into six sub-scales: Frustration, Effort, (own) Performance, Temporal Demand, Physical Demand, and Mental Demand. Participants rate each of these from 0 (low) to 100 (high) and their scores are rounded to the nearest unit of five. Participants are then given a binary choice between each pair of the six sub-scales and asked to pick which one contributes the most to the workload of the task. These fifteen binary choices are combined with the six ratings and a weighted score is derived.

### Focus group

Our focus group had five attendees, plus a moderator. Each of Algorithms I to V were presented to the group. Participants were given printed copies of MView visualisations of the proteins/MSAs under consideration with no colour scheme. Audio was recorded and transcribed.

A *scissor-and-sort* approach to the content analysis of the focus group transcript was used, as it is efficient, quick, and cost-effective [34]. We developed a categorisation system of five parts: aesthetic judgements, project judgements, analytic judgements, psychoacoustic judgements, and suggestions for future work. We then selected representative statements for each of these topics and created an interpretation of their meaning.

## Results

### Questionnaire

Our questionnaire drew five participants. This is comparable to return rates for such highly specialised questionnaire content in other sonification studies [35–37]. Our interpretation of the questionnaire returns is primarily based on qualitative rather than quantitative analysis. Thus, our questionnaire results for the present work are similar in nature and scope to a focus group.

All participants reported a high-level of experience with biological sequence data (postdoctoral study or beyond). The expertise of these respondents gives us faith that their feedback is useful and relevant to draw conclusions on the effectiveness of our sonifications. Their musical experience varied from none to undergraduate level (two to four years), which mitigates the obscuring influence of *trained ears* or great musical expertise to our assessment.

#### Task 1

In response to the first task concerning Algorithm I, all participants agreed that the sonification aided in finding the AAR. When asked what was the best thing about the sonification, participants responded that the sonification “makes repeated patterns obvious” and that it was “easier to notice repetitive sequences from repetitive sound than from eyeballing letters”. When asked about the worst thing about the sonification, participants complained of the lack of “a way to navigate the sound file easily” and that it was “hard to map the location of the repeated sound to the actual sequence”. They also noted that it “took a while to listen to” compared to looking at the sequence.

#### Task 2

In response to the second task concerning Algorithm V, all participants disagreed that the sonification helped them identify the conserved domains. When asked about the best thing about the sonification, they responded that the sonification “made gaps in the alignment obvious”, was a “complementary way of representing data”, “did help me identify the third domain”, and that it was “broadly possible to identify more conserved regions by paying attention to the volume”. In response to the worst thing about the sonification, participants responded that “it was very unpleasant to listen to”, “hard to hear conserved areas” and that “it was difficult to keep track of my place in the sequence”.

The participant’s NASA-TLX scores from the second task (Table 2) show that the participants considered *mental demand, effort,* and *frustration* as the most important contributing factors to the workload of this task. *Physical* demand was deemed by far the smallest contributor to the workload of the task. In comparison to over 780 published TLX results, the global workload score of this task lay in the 6th decile [33].

**Table 2:** NASA Task Load Index (TLX) results from task 2 of the questionnaire, composed of five responses

Factor	Raw TLX (unweighted)	NASA-TLX (weighted)
*NASA-TLX workload scores*		
Physical demand	7	3
Mental demand	75	96
Temporal demand	32	25
Performance	74	51
Effort	60	63
Frustration	56	76
Global score (Mean)	50.7	52.4
SD	26.5	33.9

Participants ranked each subscale out of 100 to give the Raw TLX. They were asked 15 binary questions comparing each subscale and selecting the most important, which gives the weights for the NASA-TLX weighted workload scores

### Focus group

The focus group comprised of two post-doctoral researchers and three PhD researchers. They came from three different countries (across Europe and North America), with three different first languages (all Indo-European). This size of focus group was appropriate to facilitate in-depth discussion [34].

The representative statements about each algorithm can be found in supplementary materials [19].

#### Project judgements

The focus group was positive about the project as a whole. “I think that with your project you need to think not about whether it is possible, because you’ve proved that it is, but can you compete against what is used nowadays”. Participants were very positive about the utility of the approach for visually impaired scientists, although all were fully sighted. Participants often remarked that a particular approach, such as the reduced alphabet in Algorithm II was a good idea, but might not be useful in every circumstance. They thought the main use of the approach was as an initial “way of filtering” their data.

#### Psychoacoustic judgements

The focus group grasped the audio metaphors for all the algorithms easily, apart from Algorithm III, wherein they found the concept of a different instrument corresponding to each letter of a reduced alphabet disrupted their following the pitch-hydrophobicity metaphor. Participants stated that they can “recognise what is present” in the moment through sound, but cannot remember it after a short time. Participants found that they had different perceptions of what aspect of the sound stood out to them most.

#### Suggestions for future work

Due to diversity of research questions, participants wanted to customize: the polarity of the sound (i.e. whether a high pitch corresponds to high or low hydrophobicity), the speed of the sonification, the navigation within the sound file, the categorisation of the sounds into step sizes, the instrumentation, and the use of reduced alphabets. They also wanted visual representation of location alongside the sonification, one participant even suggested using “one of those balls—like at karaoke”.

#### Aesthetic judgements

Varied aesthetic responses reflected the enthusiasm of the focus group—“Chaotic, but not completely chaotic” and “more diverse than expected”. Participants drew direct comparison to “scary movie” soundtracks, specifically those of John Carpenter. One participant wisecracked about “easy-listening proteins”. Algorithm V provoked the strongest responses, described as “the weirdest sound”, “doesn’t conform to the normal structure of music”, and “like someone bashing at notes”. All of these were followed by a more in depth and positive response to the sound.

#### Analytic judgements

When listening to the sonification made using Algorithm I, the same one used as the first task in the questionnaire, all participants agreed that they could “really hear it”. They engaged in what sociologist of sonification Alexandra Supper calls *sonification karaoke* by singing what they heard to be relevant [38]. This conveys the enthusiasm of the participants for the method and excitement at hearing the motif.

Responding to the reduced alphabet sonification produced using Algorithm II, the focus group found it hard to distinguish between the narrow pitch range.

Algorithm III used instruments to represent the reduced alphabet mapping, while pitches still map to the hydrophobicity scale. Participants agreed that different instruments communicated the different reduced alphabet groups very clearly, however hearing the difference in pitch within the instruments was much harder. The group felt that the reduced alphabet was meant to simplify the sound for the listener, but including the hydrophobicity pitches undid that simplification.

The participants felt that the high-level entropy sonification Algorithm IV made it “easy to discern between the highly conserved and not highly conserved regions”. However, participants also agreed that it was not easy to understand the sound in between these regions.

Participants found the sonification made with Algorithm V less clear than the others. They agreed that using the sonification as the only source of information was difficult and that they could not tell what they were listening to without a location indicator or a visual accompaniment. Participants stated that they could use this “just to get an initial idea” of the alignment.

## Discussion

Participants wanted customisability of the sonification algorithms, so they can tailor the implementation for different tasks. Positive feedback for each algorithm generally pertained to a specific task, and none were seen to be useful across a wide range of tasks on their own. Customisation would also allow users to tailor sonifications to their own psychoacoustic judgements, associating salient features to the aspects of sound they find most prominent.

User control of sequence navigation is another desirable improvement. Increased user control should reduce *frustration*, one of the larger contributors to the NASA TLX workload of the task.

Animated visualisation was recommended to give users a clear idea of location of the within the sequence or MSA during the sonification. This would reduce the *mental demand* of the task, judged to be the greatest contributor to workload by the NASA TLX assessment, at the expense of applications for scientists with visual impairments.

The development of a web-hosted tool will facilitate these improvements in visualisation, customisation, and user control.

Animation and video are important for communication of increasingly complicated scientific ideas, improving peer-to-peer communication, and inspiring public engagement [39]. However, the production of high quality audiovisual content requires significant time and effort [7]. Sonification algorithms can create compelling and scientifically accurate materials easily, reducing the outlay in production. Our feedback showed enthusiasm for sonification in the realm of public engagement and science communication. Methods developed for analysis purposes can be used for public engagement purposes and improved sound design will make more aesthetically pleasing sonifications. Different sound design may suit the varying media, such as live events, podcasting/radio productions, and scientific video production.

In the present work we used a small MSA to investigate Algorithms IV and V. While this was convenient for development and assessment purposes, any useful MSA sonification must be capable of sonifying much larger MSAs. We used Sonic Pi software on the project and would recommend it to anyone looking to experiment with sonification. Our use of Sonic Pi in this project continued from previous work [17]. This previous work took advantage of the low-effort sound design, ease of use, interactivity, and fun of Sonic Pi for public engagement purposes. However, Sonic Pi is not suited for large volumes of data. Future software in this project should utilise MIDI protocols to synthesise sound internally, thereby catering for large volumes of data, allowing for more sophisticated sound design, and removing current need for a manual step by the user. This will make better, more user-friendly software.

The adoption of the overview/detail approach used to deal with complexity in visualisations would help minimise the length of sonification [7]. This dynamic approach would deal with complexity issues by allowing users to focus down to a higher complexity sonification on their specific region of interest, while also allowing broader scale investigation of the context of that region.

Integration of sonification methods with existing sequence viewers may provide the basis for visualisation and has been suggested as a future path for protein sequence sonification [13]. On one hand, the use of existing visualisation tools may hinder benefits of the technology for visually impaired researchers. On the other hand, this approach may improve accessibility to those visualisation tools for visually impaired researchers.

While our research includes a small sample size, it was sufficient to propose future directions of research. We found that aesthetic judgements and psychoacoustic judgements were not consistent throughout our focus group. Diversity of background may be a factor in this. Taking a pragmatist aesthetic approach, we accept that experience will not be universal, but that we must design in a way to maximise meaningful dialogue with our sonifications [40]. However, taking on board the maxim that “if the key to good usability engineering is evaluation, then the key to good aesthetic interaction design is understanding how the user makes sense of the artefact and his/her interactions with it at emotional, sensual, and intellectual levels” [41], we suggest that our effectiveness assessment process attempts both aims—of evaluation and of understanding the user’s response to the sonification. Once refinements suggested by the proposed research have been implemented, qualitative and quantitative analysis with a larger sample size may be particularly valuable [42].

Our investigation has dealt with sequences that have a specific residue at each position in each sequence. In practice, problems facing sequence data visualisations include dealing with error and uncertainty, and also variant analysis [7]. This is a future possibility for sonification research implemented alongside sequence visualisation. Here sound features such as tone and roughness may be used to indicate the uncertainty in a given residue. This could be implemented in addition to current work.

Our effectiveness assessment proposed clear improvements to the ways in which users can interact with the sonification. The assessment of software effectiveness by investigating the user experience has long been key to commercial software development [43] but is not generally included in publications on visualisation software for bioinformatics. For example, we found no mention of effectiveness or usability assessment processes in articles about individual software tools, such as Tablet (a next-generation sequencing data viewer) [44], Artemis (a high-throughput sequencing visualisation tool) [45], and Aliview (an MSA browser) [46]. We find the same absence in review papers, such as a 2010 review of visualisation of MSAs, phylogenies, and gene family evolution [9], a 2020 review of visualisation tools for human structural variations [47], and many reviews of genome browsers [48–50]. Particularly now there are a large number of visualisation packages for MSAs and many other kinds of biological data, we recommend such assessments. These would make it easier to understand the effectiveness of visualisation tools and compare them with each other, and with sonification as a complementary or alternative approach.

Within the field of sonification, research often focusses on techniques for sonifying data rather than on the content and interpretation of the sonified data [51]. Qualitative research methods can shift this focus by evaluating the success of sonification techniques using the phenomenological input of end-users (domain scientists, in this case bioinformaticians), centring the context of their use in the design process.

We also carried out quantitative analysis. While evaluating novel methods for data exploration is difficult, as insight cannot be quantified in a perfectly satisfactory way, the NASA TLX is simple to implement, cost-effective, and has thirty years of research and applications behind it [52]. The six subscales provide a language to articulate nuances in the difficulty of tasks.

Future work on this project may result in tools useful for scientists with visual impairment. While feedback from people who are not visually impaired may not provide good positive evidence for the potential of this technology in this area, experimentation suggests that perception of sound polarity between sighted, blind, and visually impaired people is similar [53]. This suggests that success with sighted participants may be transferable, insofar as the perception is concerned. Future effectiveness assessment should include scientists with visual impairments to establish the utility of the approach.

The potential of sonification of protein sequence data is clear, with the twin untapped wellsprings of human psychoacoustic intuition and latent enthusiasm for the approach well placed to sustain and nurture the field as it continues to grow. If music be the food of innovative scientific research, play on.

## Conclusions

For single protein sequences, the success of our sonifications for conveying particular features was supported by both the survey and research group. The focus group showed enthusiasm for the approach.

There was more limited evidence that the multiple sequence alignment sonifications successfully conveyed information, with a higher level sonification showing more success than a finer grained sonification. Overall, the complexity of the data resulted in difficulties for a single, nonconfigurable sonification without supporting visualisation. Additional work is required to make these sonifications useful to researchers.

The qualitative feedback process centred on bioinformaticians (i.e. end-users of the sonifications) provided high-quality, contextualised, and specific recommendations on improvements to the technology. Key lessons were common for improving both MSA and single protein sequence sonifications.

## Data Availability

Website with examples—https://sonifyed.com/bmc-bioinformatics-2021. GitHub Repository of code—https://doi.org/10.5281/zenodo.4683746. The dataset and Perl scripts supporting the conclusions of this article are available in the University of Edinburgh’s DataShare repository—https://doi.org/10.7488/ds/3023. Operating system(s): Platform independent. Programming language: Perl (v5.16.3) and Sonic Pi (v3.1.0). License: Sonic Pi—Open Source Project released under the MIT Licence, Perl—GNU General Public License.

## Abbreviations

AAR: Amino acid repeat
GES: Goldman, Engelman, and Steitz hydrophobicity scale
MIDI: Musical instrument digital interface
MSA: Multiple sequence alignment
NASA: National Aeronautics and Space Administration
PMSon: Parameter-mapping sonification
TLX: Task load index
